# Repetitive mild traumatic brain injury causes synergistic effects on mortality

**DOI:** 10.17912/micropub.biology.000356

**Published:** 2021-01-14

**Authors:** Ashley M Willes, Tori R Krcmarik, Alexander E Daughtry, Douglas J Brusich

**Affiliations:** 1 University of Wisconsin - Green Bay, Green Bay, WI USA

## Abstract

Repetitive mild TBI (rmTBI) events are common in the U.S. However, rmTBI is challenging to study and this contributes to a poor understanding of mechanistic bases for disease following these injuries. We used fruit flies (*D. melanogaster*) and a modified version of the high-impact trauma (HIT) method of TBI to assess the pattern of mortality observed after rmTBI. We found that the pattern of mortality was synergistic after a critical number of injuries, similar to that observed previously at more moderate levels of TBI severity. The identity of cellular and molecular factors which contribute to the synergistic effect on mortality remain unknown, but this model offers a platform for investigation into such factors.

**Figure 1. rmTBI causes a synergistic effect on mortality:**
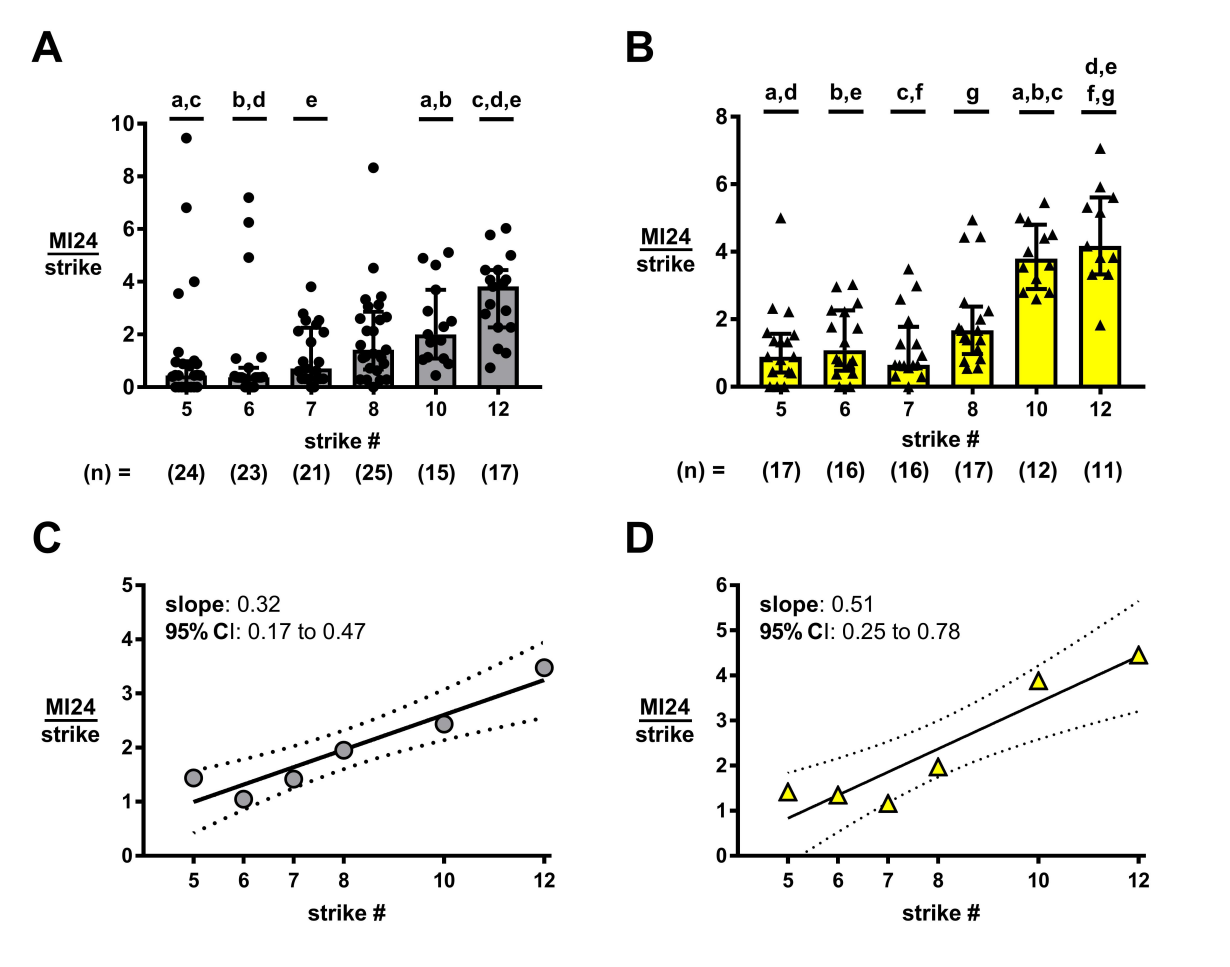
**(A,B)** Mortality per injury is greater for both *w^1118^* (A) and *y^1^w^1^* (B) flies subjected to 10 or 12 strikes vs lesser injury numbers. Data plotted are median with IQR. Indicated ‘n’ values and symbols reflect each vial of ≥ 40 flies. Datasets with shared letters are statistically different from each other (p < 0.05 by Kruskal-Wallis with Dunn’s Correction). **(C,D)** Overall MI24/strike values across 5-12 strikes for both *w^1118^* (C) and *y^1^w^1^* (D) flies best fit a positive slope line. Symbols represent the overall MI24/strike value for each condition as determined using summed count data from each vial of ≥ 35 flies. Solid lines indicate the best-fit line with 95% confidence bands plotted by dashed lines. n ≥ 710 flies per condition for *w^1118^*; n ≥ 537 flies per condition for *y^1^w^1^*.

## Description

Millions of Americans suffer traumatic brain injury (TBI) each year (Taylor *et al.* 2017). The vast majority (~90%) of TBI events in the U.S. are mild TBIs, which includes concussions (Cassidy *et al.* 2004). Mild TBI commonly causes temporary symptoms, but repetitive mild TBI (rmTBI) is associated with long-term consequences, which may take years to manifest (Bailes *et al.* 2013). Current animal models of rmTBI have drawbacks which contribute to the scarcity of evidence for mechanistic bases of dysfunction and disease.

We previously reported an extended fly (*D. melanogaster*) model of TBI based on the spring-based, high-impact trauma (HIT) method (Katzenberger *et al.* 2013; Putnam *et al.* 2019). We showed that deflection of the spring and attached vial of flies to 90° from horizontal resulted in severe injuries as evidenced by rates of mortality, but that reduced deflections (60-80°) had lesser effects (Putnam *et al.* 2019). Moreover, we showed that repetitive injuries at the relatively moderate deflections of 70° and 80°, but not severe injuries at 90° nor mild injuries at 60°, resulted in a synergistic effect on mortality (Putnam *et al.* 2019). One potentially confounding issue preventing identification of synergistic effects at 60° was the low mortality rate after the 1-4 injuries administered (Putnam *et al.* 2019). We chose to further investigate the relationship between mortality and rmTBI using the 60° deflection by extending the injury number to 5-12 strikes.

The main outcome measured using the HIT model of TBI in flies is the mortality index at 24 hours (MI24) (Katzenberger *et al.* 2013). The pattern of mortality across varied HIT numbers can be assessed by dividing each MI24 value by its respective strike number (MI24/strike) (Katzenberger *et al.* 2013; Putnam *et al.* 2019). If mortality from each strike is additive then the MI24/ strike values should be equivalent, but, if mortality is synergistic, then the MI24/strike values should become increasingly large with strike number. Neither, *w^1118^* nor *y^1^w^1^* flies showed any differences in MI24/ strike values across 5-8 strikes (Fig. 1A, 1B respectively). However, by 10 strikes both genotypes showed larger MI24/strike values than for 5 or 6 strikes, and we found a more pronounced effect at 12 strikes (Fig. 1A, 1B). To more fully assess the pattern of mortality across injury number, we checked the MI24/strike data using a line fit. When we used data across only 5-8 strikes neither *w^1118^* nor *y^1^w^1^* flies deviated from fit to a zero-slope line (*w^1118^*: p = 0.34; *y^1^w^1^*: p = 0.45), consistent with additive effects as seen previously. However, inclusion of data from 5-12 strikes for each of *w^1118^* and *y^1^w^1^* resulted in positive slope lines that significantly deviated from zero-slope lines (Fig. 1C, 1D respectively; *w^1118^*: p = 0.004; *y^1^w^1^*: p = 0.006), consistent with a synergistic relationship.

Our data show that mild TBI causes synergistic effects on mortality after a requisite number of injuries, possibly because cumulative cellular stress surpasses a critical threshold. We expect that many of the secondary injury pathways active in animals following our mild TBI (60°) methodology overlap with pathways previously reported (Katzenberger *et al.* 2015; Katzenberger *et al.* 2016; Anderson *et al.* 2018; Saikumar *et al.* 2020; Swanson, Rimkus, *et al.* 2020; Swanson, Trujillo, *et al.* 2020). However, the identity of any specific factor(s) which set or scale the sensitivity to rmTBI remain unknown. Identification of such factor(s) may allow us to develop diagnostic tools more sensitive to tracking rmTBI outcomes and lead to identification of genetic risk factors which contribute to disease following rmTBI.

## Methods

**Fly Husbandry and TBI Methodology**

Stocks of *w^1118^* (BL 5905) and *y^1^w^1^* (BL 1495) were obtained from the Bloomington Drosophila Stock Center (Bloomington, Indiana, USA). Flies were maintained in a humidified 25°C incubator, on a 12H:12H light:dark cycle, and on a standard glucose-cornmeal-yeast food (Putnam *et al.* 2019).

Methods for TBI were based on those reported previously (Katzenberger *et al.* 2013; Putnam *et al.* 2019). Briefly, flies were collected using CO_2_ anesthesia (35 – 60 flies per vial), and subjected to TBI by 5 days after eclosion. TBI was administered using a modified high-impact trauma (HIT) device with a stopping point which limited the spring deflection to 60°. The vial was released and allowed to collide with a foam pad covered by a 1/16” rubber pad. Injuries were spaced 15 seconds apart and repeated until a total of 5-12 injuries were administered. Animals were hand-transferred to food vials and returned to the 25°C incubator until they were assessed for mortality 24 hours later. The mortality index at 24 hours (MI24) was calculated as: MI24 = number of flies dead at 24 hours/total number of flies * 100. MI24/ strike values were calculated by dividing the MI24 value by the administered strike number.

**Statistics**

MI24/strike values for comparisons of mean ranks across 5-12 strikes were calculated for each vial of at least 40 flies. Conditions were compared by Kruskal-Wallis with multiple comparisons of mean ranks and Dunn’s correction at the level of α = 0.05 (GraphPad Prism 7).

The pattern of mortality was tested via line-fit of MI24/strike data across strike number. A single MI24 value for each condition was calculated from summed dead:total count data from each vial of at least 35 flies. This overall MI24 was then divided by the strike number to calculate the MI24/strike value for each condition. MI24/strike data points were then plotted across strike number using the linear fit mode in the nonlinear regression analysis toolkit, fitted using the least squares fit mode, compared to a hypothetical slope of zero via the extra sum-of-squares F test at a level of α = 0.05, and plotted with the best-fit slope and the asymmetrically determined 95% confidence interval (CI) (GraphPad Prism 7).
